# Effects of a fibre-enriched milk drink on insulin and glucose levels in healthy subjects

**DOI:** 10.1186/1475-2891-8-45

**Published:** 2009-10-01

**Authors:** Netta Lummela, Riina A Kekkonen, Tiina Jauhiainen, Taru K Pilvi, Tuula Tuure, Salme Järvenpää, Johan G Eriksson, Riitta Korpela

**Affiliations:** 1 Valio Ltd, Research Center, PO Box 30, 00039 Valio, Finland; 2 University of Helsinki, Institute of Biomedicine, PO Box 63, 00014 University of Helsinki, Finland; 3 Medcare Foundation, Hämeentie 1, 44100 Äänekoski, Finland; 4 University of Helsinki, Department of General Practice and Primary Health Care, PO Box 20, 00014 University of Helsinki, Finland; 5 National Institute for Health and Welfare, Mannerheimintie 166, 00300 Helsinki, Finland; 6 Helsinki University Central Hospital, Unit of General Practice, Helsinki, Finland

## Abstract

**Background:**

The glycaemic response to foods is dependent on the quality and content of carbohydrates. Carbohydrates in the form of dietary fibre have favourable effects on insulin and glucose metabolism and may help to control energy intake. Dairy products have a relatively low carbohydrate content, and most of the carbohydrate is in the form of lactose which causes gastrointestinal symptoms in part of the population. In order to avoid these symptoms, dairy products can be replaced with lactose-free dairy products which are on the market in many parts of the world. However, the effects of lactose-free products on insulin and glucose metabolism have not been studied.

**Methods:**

In the present study, we investigated the effects of 1) a lactose-free milk drink, 2) a novel fibre-enriched, fat- and lactose-free milk drink and 3) normal fat-free milk on serum glucose and insulin levels and satiety using a randomized block design. Following an overnight fast, 26 healthy volunteers ingested 200 ml of one of these drinks on three non-consecutive days. Insulin and glucose levels and subjective satiety ratings were measured before the ingestion of the milk product and 20, 40, 60, 120 and 180 minutes after ingestion. The responses were calculated as the area under the curve subtracted by the baseline value (AUC minus baseline).

**Results:**

The insulin response was significantly lower for the fibre-enriched milk drink than it was for the other milk products (AUC, *P *= 0.007). There were no differences in the response for glucose or in the AUC for the subjective satiety ratings between the studied milk products.

**Conclusion:**

The present results suggest that this novel milk drink could have positive effects on insulin response.

## Background

A combination of metabolic disturbances, including central obesity, cardiovascular disease, dyslipidaemia, insulin resistance and elevated blood pressure, affects the life of a quarter of the world's adult population [1]. Continuous imbalance in the above-mentioned metabolic conditions, in association with obesity, leads to an increased risk of developing type 2 diabetes and related metabolic co-morbidities. Recent studies have shown that lifestyle changes have promising effects on the prevention of type 2 diabetes [2-6]. Weight loss by dietary modification and increased physical activity is the primary recommendation also in the treatment of the metabolic syndrome [7,8]. Generally, maintaining a balance between energy intake and expenditure has proven difficult for people struggling with overweight. The composition of the diet and the satiating effects of foods have emerged as an important research area in the field of overweight and glucose and insulin metabolism.

An inverse association between the consumption of dairy products and BMI has been detected in several study populations [9-12], and the increased intake of dairy products has been shown to decrease weight gain [13]. The effect of dairy products on weight loss remains a controversial area of research [14]. Epidemiological studies have indicated that an increased intake of calcium and dairy products may be associated with a lower prevalence of the metabolic syndrome and type 2 diabetes [15-18]. Thus, dairy products could be considered as a component of diets aiming at optimal weight management.

The prevalence of lactose-intolerance differs among European populations, ranging from 4% (Denmark) to 50% (average prevalence in Italy) [19]. Gastrointestinal symptoms may reduce the intake of dairy products and people who are not able to consume dairy products may be at risk for inadequate calcium and vitamin D intake[9]. Lactose-free products are critical for assuring adequate calcium and vitamin D intake in lactose-intolerant people. In the digestive process, the lactose-molecule is split into two monosaccharides, glucose and galactose, which may have independent effects on insulin metabolism. This should be taken into consideration when potential consumers include people with overweight, insulin resistance and lactose-intolerance. To our knowledge, the insulin responses of lactose-free dairy products have not been previously studied. Moreover, the effects of a lactose-free milk drink on glucose metabolism are not known.

The glycaemic response to particular foods depends on several variables, including the structure of the carbohydrate, the fibre content and the preparation or processing of the food in question [20]. Foods with a low-glycaemic response have been shown to exert beneficial effects on blood glucose and insulin levels [21,22], on the incidence of type 2 diabetes [23,24] and on prolonged satiety [25]. Therefore, it is possible that low-glycaemic foods contribute to preventing the development of metabolic disorders, such as insulin resistance, dyslipidaemia and cardiovascular diseases [26,27]. However, the current data are inconsistent [28].

Increasing fibre and whole grain intake affects glucose and insulin metabolism favourably and may enhance insulin sensitivity in obese subjects and in subjects with impaired glucose tolerance [29-33]. Among other functions, fibre enhances satiation and satiety due to its viscosity-producing and bulking effects [34] and it may promote weight reduction in obese subjects [35]. The effects of natural, added, soluble and insoluble fibres on weight reduction have been found to be fairly similar [35]. Adding soluble fibre in fat-free dairy products could enhance the products' positive effects on weight management and balance the otherwise high insulin response of dairy products.

The aim of the present study was to compare the acute effects of a lactose-free milk drink, a novel milk drink (fibre-enriched, fat- and lactose-free) and regular fat-free milk on fasting insulin and glucose levels and on subjective satiety levels in healthy subjects.

## Methods

### Subjects

Thirty-three subjects were recruited by an advertisement in a local newspaper. Healthy men and women within the age range of 18-65 years, with normal and regular eating habits and a body mass index (BMI) of 20-30 kg/m^2 ^were eligible for the study. Subjects who were diabetic or had a fasting serum glucose level of 6.2 mmol/l or more, had milk protein allergy or lactose intolerance, were pregnant or lactating or took medication for chronic digestive disorders, or were participating in another study, were not eligible for the present study. Two subjects were excluded because of their medical history and five discontinued the study for personal reasons. Twenty-six healthy subjects (16 female and 10 males completed the study. The subjects' mean age was 48 ± 11 years (age range 25-64) and their mean BMI was 24.6 ± 2.9 kg/m^2 ^(BMI range 20-30).

### Study protocol

A randomized response profile block design study was conducted to investigate the effect of different milk products on serum glucose levels, insulin levels and satiety. Prior to the intervention, all enrolled subjects underwent an interview and their lifestyle habits (medical history, smoking, alcohol use and physical activity) were assessed. The subjects' waist and hip circumference and height and body weight without shoes and outdoor clothing were measured, and their BMI was calculated using the standard equation (kg/m^2^). Furthermore, cholesterol (enzymatically), triglyceride (enzymatically) and glucose concentrations were measured to assess baseline values.

The intervention comprised three study days. The subjects were advised to avoid exhausting exercise in the evening before a study day. After a 12-hour overnight fast, a venous blood sample was collected and the subjects consumed the study product in 20 minutes. Additional blood samples were taken 20, 40, 60, 120 and 180 minutes after the fasting blood sample. The subjective satiety level was measured immediately after each sampling. After 180 minutes, the subjects were allowed to eat freely, but they were instructed to keep a record of everything they ate during the rest of the day in a food diary. The subjects gave their written informed consent before entering the study. The study protocol was approved by the Ethics Committee of the Hospital District of Helsinki and Uusimaa.

### Study products

The studied milk products comprised fat-free milk, a fat- and lactose-free milk drink and a very low-energy polydextrose and calcium-enriched fat- and lactose-free milk drink (fibre-enriched milk drink) (Valio ProFeel^®^, Valio Ltd, Helsinki, Finland). The subjects consumed 200 ml of one of these products on three non-consecutive study days. The energy and nutrient contents of the milk products are shown in Table 1. As illustrated, the fibre-enriched milk-drink had the highest calcium content (180 mg/100 g). The vitamin D content of all the studied products was 0.5 μg/100 g. The fibre enrichment did not affect the taste of the milk drink, which was confirmed by a blinded taste test performed by expert panellists.

**Table 1:** Nutrient composition in 100 g of the studied products.

	**Fibre-enriched** **milk drink**	**Lactose-free milk drink**	**Fat-free milk**
Energy (kJ)	84	110	140
Protein (g)	3.3	3.3	3.3
Carbohydrate (g)	1.8	3.1	4.9
of which lactose (g)	0.0	0.0	4.9
Fibre (g)	1.5	0.0	0.0
Fat (g)	0.0	0.0	0.0
Calcium (mg)	180	120	120

### Blood measurements

The venous blood samples were taken into gel serum tubes and centrifuged (10 min at 3000 rpm) within 30 minutes of sampling. The serum was immediately separated and frozen in -20°C for further analysis of glucose and insulin levels. Serum insulin was analysed using luminoimmunometric techniques and glucose was analysed using hexokinase techniques at United Laboratories Ltd, Helsinki, Finland.

### Subjective satiety measurements

Subjective satiety was measured by Visual Analogue Scale (VAS) and Satiety Labeled Intensity Magnitude (SLIM) ratings. The following VAS ratings were used: 'How hungry do you feel right now?' (hunger), 'How full do you feel right now?' (fullness), 'How much do you think you could eat right now?' (prospective food consumption), 'How strong is your desire to eat right now?' (desire to eat) and 'How satisfying did you find the study product?' (satisfying satiety effect of the study product) were used. The VAS consisted of 100 mm horizontal lines with extreme sensations anchored at the ends of the lines. The subjects completed the VAS questionnaire by placing a vertical slash on the line corresponding to their sensation. SLIM consisted of one question, 'How hungry or full are you right now?', with a vertical line scale for subjects to mark horizontally to indicate their potential sensations of fullness and hunger. Subjective satiety rating questionnaires were completed immediately after each blood sampling.

### Food diaries

The subjects completed a food diary on all three study days. They were advised to mark down, as precisely as possible, what was consumed during a study day, as well as the time and place of consumption. The subjects used grams or decilitres to specify the amount of food consumed and they were supplied with a Portion Size Booklet (National Public Health Institute, University of Helsinki, Department of Nutrition, Helsinki 1985) to help them estimate the amounts and portion sizes. The food diaries were analysed with Nutrica 3.1 software programme (KELA, Turku, Finland).

### Statistical analyses

The results are presented as means with standard deviations (SD) and 95% confidence intervals (CI). The responses to insulin and glucose levels and subjective satiety were calculated as the area under the curve subtracted by the baseline value (AUC minus baseline). The tested products were compared with ANOVA for repeated measures. Sidak's adjustment was applied to correct levels of significance for post hoc pairwise testing. The normality of variables was evaluated using the Shapiro-Wilk statistics. Insulin values under the detection limit 2.0 mU/l were compensated with value 1.0 mU/l (28%). Statistical analyses were performed with SPSS 14.0 software (SPSS Inc., Chicago, Illinois, USA).

## Results

### Glucose and insulin

**Figure 1:**
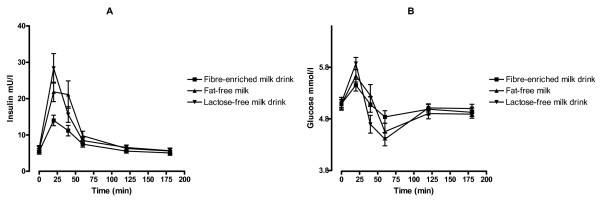
**(A) Changes from fasting values for insulin responses following the ingestion of a fibre-enriched milk drink (black square), fat-free milk (black triangle up) and a fat- and lactose-free milk drink (black triangle down)**. (B) Changes from fasting values for glucose responses following the ingestion of a fibre-enriched milk drink (black square), fat-free milk (black triangle up) and a fat- and lactose-free milk-drink (black triangle down). Values are means with 95% confidence intervals.

There was a statistically significant difference in AUC minus baseline for insulin between the milk products (*P *= 0.007). In a pairwise comparison, serum insulin concentration increased significantly less following the consumption of the fibre-enriched milk drink compared to fat-free milk and the fat-and lactose-free milk drink (2.1 vs. 4.3, *P *= 0.036 and 2.1 vs. 3.9, *P *= 0.011, respectively) (Fig. 1(B)). There were no differences in AUC minus baseline for glucose between the milk products (*P *= 0.31). The glucose curves for milk products are presented in Fig. 1(A).

### Satiety and food intake

No significant differences were observed in AUC minus baseline values for subjective satiety ratings (Table 2). The fat- and lactose-free milk drink was found to be more satiating than the fibre-enriched milk drink at 20 minutes after consumption (48.8 vs. 36.1, *P *= 0.042). Based on the food diaries, the subjects' energy, protein, total fat, fatty acid and fibre consumption remained the same throughout the study and did not differ significantly between the three study days. Carbohydrate consumption was higher on the fibre-enriched milk drink study day compared to the fat-free milk study day (*P *= 0.03) (Table 3).

**Table 2:** AUC minus baseline for subjective satiety ratings 180 minutes after ingestion of tested products (n = 26).

**Satiety rating**	**Study day with**						*P*-value*
		
	**Fibre-enriched milk drink**		**Fat-free milk**		**Lactose-free milk drink**		
	**Mean**	**SD**	**Mean**	**SD**	**Mean**	**SD**	
Hunger	0.8	20.4	1.0	15.7	3.7	16.1	0.73
Desire to eat	1.0	19.9	1.3	14.8	2.3	13.9	0.94
Fullness	7.4	15.0	5.3	10.8	6.4	14.8	0.79
Prospective food consumption	1.1	15.7	3.3	13.5	4.5	9.8	0.63
SLIM	0.7	12.7	-1.3	10.5	-4.6	9.7	0.14

SLIM, Satiety Labeled Intensity Magnitude for hunger and fullness

*ANOVA for repeated measurements

**Table 3:** Energy, macronutrient and fibre intake during the three study days (n = 25).

	**Study day with**						*P*-value*
		
	**Fibre-enriched milk drink**		**Fat-free milk**		**Lactose-free milk drink**		
	**Mean**	**SD**	**Mean**	**SD**	**Mean**	**SD**	
Energy (kJ)	7767	2572	7041	1732	6707	1985	0.10
Protein (g)	84.2	23.6	83.0	23.2	75.7	22.4	0.25
Carbohydrates (g)	231^†^	86	197^‡^	49	195	68	0.033
Total fat (g)	58.5	29.0	54.8	22.5	49.2	19.6	0.26
SAFA (g)	25.8	14.1	24.8	12.5	22.4	10.1	0.36
PUFA (g)	9.5	6.3	7.1	3.4	7.0	3.2	0.093
MUFA (g)	17.1	9.8	16.1	8.6	15.4	8.3	0.78
Fibre (g)	30.6	21.3	24.8	12.9	26.5	16.4	0.28

SAFA, saturated fatty acids; PUFA, polyunsaturated fatty acids; MUFA, monounsaturated fatty acids

*ANOVA for repeated measurements

^†,‡^Mean values within a row with unlike superscript letters are significantly different (*P *< 0.05).

## Discussion

In this study, we investigated the effects of a lactose-free milk drink, a novel fibre-enriched lactose- and fat-free milk drink and regular fat-free milk on glucose and insulin responses and satiety levels in healthy subjects. The insulin response was markedly lower after the consumption of the fibre-enriched milk-drink compared to the other products. Although there was no significant difference in the AUC response of glucose between the study products, the fibre-enriched milk drink appeared to equalize the glucose response.

The metabolic syndrome is characterized by insulin resistance and hyperinsulinemia, as well as several detrimental symptoms, such as hypertension, fatty liver disease and impaired glucose disposal [36]. In addition, continuously elevated insulin concentrations impair normal vascular function [37]. Although the effect of the novel milk-drink on glucose response did not differ from the other studied drinks, it produced a significantly lower postprandial insulin concentration. The novel milk drink has the lowest carbohydrate content, which can partly explain the lower insulin response. Another potential explanation for the lower insulin response is the fibre added in the milk drink. Polydextrose, the soluble fibre used in the present study, is a polysaccharide, but it has physiologic effects comparable to other dietary fibres [38]. In several studies, fibre-rich foods have been shown to lower insulin responses in healthy and diabetic subjects [39-45]. On the other hand, Gruendel *et al*. observed that when 5 or 10 g of insoluble carob fibre are consumed with a glucose drink, the insulin response increases compared to drinking only a glucose drink [46]. To our knowledge, the effect of fibre enrichment in milk on insulin levels has not been studied before. Because the milk carbohydrate, lactose, is totally removed from the novel milk drink using the chromatographic technique, the energy and monosaccharide content of the milk drink is further reduced. Therefore, in addition to the added polydextrose, the low-energy and low-carbohydrate content of the milk drink have most likely contributed to the observed effect. Epidemiological studies have shown high vitamin D status to be inversely associated with insulin resistance [47]. Regardless of this interesting finding, it is unlikely that the vitamin D content of the drink contributed to the lower insulin response in this study, since all three study drinks contained the same amount of vitamin D.

The present study does not support the earlier studies which indicate that fibre and fibre-enriched products stabilise postprandial glucose levels [22,39,40,44,45]. However, a trend towards a more balanced glucose response was observed after the consumption of the fibre-enriched milk drink. Generally, dairy products have a low glycaemic response [48], which is due to their low carbohydrate and high protein content [49]. The fibre enrichment and the low calorie and carbohydrate content of the milk drink might explain the shape of the glucose curve. Furthermore, the lack of significant difference in the postprandial glucose response could also be due to the selection of healthy, glucose-tolerant individuals who show only minor fluctuation in glucose levels due to a well-preserved insulin secretion capacity.

Dietary fibre may enhance satiety by slowing gastric emptying and by delaying absorption [35]. Soluble fibres absorb water, form a gel and may increase abdominal distension, which creates a sensation of fullness [34,50]. We used a Visual Analogue Scale (VAS) questionnaire to study the effect of milk products on satiety, because it is the most commonly used satiety measurement and makes comparison to other studies possible [51]. Another rating scale, Satiety Labeled Intensity Magnitude (SLIM) [52], was also used. In the present study, fibre enrichment did not increase satiety. Furthermore, neither differences in the energy intake at the subsequent breakfast meals or in the energy intake during the rest of the study day were found. The reason for not finding a satiating effect in this study might be due to the moderate amount of fibre in the study product and the fairly small amount of product consumed. In a study by King *et al*. [53], the effects of polydextrose and xylitol on appetite were examined independently and together with a greater amount of fibre than in this study. King *et al*. used 25 g of polydextrose or 25 g of xylitol or 12.5 g of polydextrose and 12.5 g of xylitol. In the present study, the amount of polydextrose was only 3 g. In the study by King *et al*., the combination of the studied carbohydrates significantly increased the feeling of fullness. Used alone, polydextrose significantly suppressed food intake compared to a control product. Similarly, another soluble fibre (oligofructose, 16 g/day) significantly reduced energy intake, hunger and prospective food consumption and increased satiety compared to placebo treatment [54]. The products studied in the present study were beverages, which may have different effects on satiety compared to solid foods [55].

## Conclusion

A fibre-enriched fat-free milk drink with a low carbohydrate content helped to balance the postprandial insulin response. The insulin lowering effect of the fibre-enriched milk drink warrants further investigation in people suffering from impaired glucose regulation. More research is also needed to establish whether the changes in insulin response occur when the milk-drink is consumed with a whole meal and also to investigate the long term effects of fibre-enriched dairy products on glucose and insulin metabolism.

## Competing interests

The study has been funded by Valio Ltd. and the Finnish Funding Agency for Technology and Innovation. Netta Lummela, Riina Kekkonen, Tiina Jauhiainen, Taru Pilvi, Tuula Tuure and Riitta Korpela are employees at Valio Ltd Research Centre. None of the authors had any conflicts of interest.

## Authors' contributions

NL participated in planning the study and coordinated the study, created the database, participated in interpreting the statistical results and wrote the paper. RAK designed the protocol, participated in interpreting the statistical results and helped in writing the manuscript. TJ designed the protocol and revised the manuscript. TT helped to design the protocol and revised the manuscript. SJ performed the statistical analysis and revised the manuscript. JE participated in planning the study, supervised the study as MD and revised the manuscript. RK participated in planning the study, was responsible for the management of the study and revised the manuscript. All authors have read and approved the final manuscript.
